# Inequalities in multiple health outcomes by education, sex, and race in 93 US counties: Why we should measure them all

**DOI:** 10.1186/1475-9276-13-47

**Published:** 2014-06-13

**Authors:** Yukiko Asada, Alyce Whipp, David Kindig, Beverly Billard, Barbara Rudolph

**Affiliations:** 1 Department of Community Health and Epidemiology, Dalhousie University, 5790 University Avenue, Halifax, Nova Scotia B3H 1V7, Canada; 2 Department of Population Health Sciences, University of Wisconsin Medical School, 707 WARF Building, 610 North Walnut Street, Madison, Wisconsin 53726, USA; 3 Center for Health Systems Research and Analysis, University of Wisconsin-Madison, 610 North Walnut Street, WARF Building, 11th Floor, Madison, Wisconsin 53726-2397, USA

**Keywords:** Health inequalities, Public health, Health status, Health policy

## Abstract

**Introduction:**

Regular reporting of health inequalities is essential to monitoring progress of efforts to reduce health inequalities. While reporting of population health became increasingly common, reporting of a subpopulation group breakdown of each indicator of the health of the population is rarely a standard practice. This study reports education-, sex-, and race-related inequalities in four health outcomes in each of the selected 93 counties in the United States in a systematic and comparable manner.

**Methods:**

This study is a cross-sectional analysis of large, publicly available data, 2008, 2009, and 2010 Behavioral Risk Factor Surveillance System (BRFSS) Selected Metropolitan/Micropolitan Area Risk Trends (SMART) and 2008, 2009, and 2010 United States Birth Records from the National Vital Statistics System. The study population is American adults older than 25 years of age residing in the selected 93 counties, representing about 30% of the US population, roughly equally covering all geographic regions of the country. Main outcome measures are: (1) Attribute (group characteristic)-specific inequality: education-, sex-, or race-specific inequality in each of the four health outcomes (poor or fair health, poor physical health days, poor mental health days, and low birthweight) in each county; (2) Overall inequality: the average of these three attribute-specific inequalities for each health outcome in each county; and (3) Summary inequality in total morbidity: the weighted average of the overall inequalities across the four health outcomes in each county.

**Results:**

The range of inequality across the counties differed considerably by health outcome; inequality in poor or fair health had the widest range and the highest median among inequalities in all health outcomes. In more than 70% of the counties, education-specific inequality was the largest in all health outcomes except for low birthweight.

**Conclusions:**

It is feasible to extend population health reporting to include reporting of a subpopulation group breakdown of each indicator of the health of the population at a small jurisdictional level using publicly available data. No single group characteristic or health outcome represents the whole picture of health inequalities in a population. Examining multiple group characteristics and outcomes in a comparable manner is essential in reporting health inequalities.

## Introduction

Health inequalities continue to be one of the critical challenges of our times. Reducing health inequalities as well as improving overall health are now considered as twin goals of population health in many countries and sub-national jurisdictions [1]. Globally, increasing inequality is identified as one of the five shifts of contemporary issues since 2000, when the Millennium Development Goals (MDGs) were set for 2000–2015, and considerations for inequalities in general and in health care and health domains are prominent in efforts to identify post-MDGs, Sustainable Development Goals for 2015–2030 [2].

Regular reporting of health inequalities is essential to monitoring progress of, or lack thereof, efforts to reduce health inequalities [3,4]. While reporting of population health has become increasingly common and sophisticated [5,6], reporting of a subpopulation group breakdown of each indicator of the health of the population (e.g., life expectancy or disease prevalence rates) remains challenging. Major hindrances to regular reporting of health inequalities are a myriad of information and data limitation. Although each jurisdiction has historical and social reasons to be concerned about health inequality associated across a certain group characteristic (e.g., race in the United States and social class in the United Kingdom), health inequalities extend to many group characteristics, reflecting complex causal pathways of multiple determinants of health [7,8]. By measuring, for example, income-, education-, sex-, race-, and geography-related inequalities in various health outcomes included in a population report, we are presented with numerous information and faced with a challenge of reporting it systematically and comparably. In addition, data that allow breakdown of many health outcomes by multiple group characteristics are often limited, especially at a small jurisdictional level or in low and middle income countries.

By addressing these methodological and data challenges, in this paper we report education-, sex-, and race-related inequalities in four health outcomes (poor or fair health, poor physical health days, poor mental health days, and low birthweight) for each of the 93 counties in the United States selected as a convenience sample. We use large, publicly available data sources and apply a measurement approach that nests inequalities in a number of health outcomes by multiple groups in a logical manner. Our analysis demonstrates the feasibility of extending population health reporting from overall health to health inequalities using publicly available data sources and underscores the importance of examining a manifold of health inequality information in a comparable manner.

## Methods

### Data

We used a pooled 2008, 2009, and 2010 Behavioral Risk Factor Surveillance System (BRFSS) Selected Metropolitan/Micropolitan Area Risk Trends (SMART) [9] and a pooled 2008, 2009, and 2010 United States Birth Records from the National Vital Statistics System (NVSS) [10].

#### BRFSS SMART

The BRFSS is an annual random-digit-dialed phone survey of a nationally representative sample of American adults, designed by the Centers for Disease Control and Prevention (CDC) and administered monthly by the 50 state health departments and the District of Columbia. It collects information on health conditions and risk behaviors, mainly for state-level analysis. SMART is a subset of the BRFSS and offers information on selected Metropolitan/Micropolitan Statistical Areas (MMSAs) with at least 500 respondents and, within those MMSAs, counties with at least 250 respondents. The response rates were 34.4% (2008), 34.9% (2009), and 35.8% (2010).

#### NVSS Birth records

The NVSS, housed in the National Center for Health Statistics, maintains a national registry of births, deaths, marriages, divorces, etc. mandated by federal and state laws. We obtained 2008–2010 birth records through the CDC Wonder website [11].

#### Selection of counties and data years

BRFSS SMART had information on 266, 283, and 302 counties for 2008, 2009, and 2010, respectively, and NVSS Birth Records datasets had information on 524 counties for these same years. We selected 93 counties by balancing considerations for adequate sample sizes and minimum pooling of data years. Specifically, to be included in our analysis, respondents in BRFSS SMART must have age information (for age-standardization of health outcome variables) and be older than 25 years of age (for reliable calculation of education). In addition, the county must appear in both BRFSS SMART and NVSS Birth Records datasets and in every data year used for the analysis (200 counties). Furthermore, the county must have adequate sample size for each group of each attribute used for calculating inequalities (93 counties). Following a conservative convention of data suppression, we set 50 observations, that is, 50 respondents (BRFSS SMART) or births (NVSS Birth Records), as the minimum sample size. We collapsed groups when a group had less than 50 observations (e.g., black and other groups for race). But to calculate inequality, each attribute needed to have at least two groups, both of which must have at least 50 observations. We pooled three years of data to address the sample size issue. The selected 93 counties represent about 30% of the US population, roughly equally covering all geographic regions of the country.

### Variables

#### Health outcomes

We used four measures of morbidity used in the County Health Rankings and Roadmaps initiative (CHR), which ranks most of the counties in the US according to their performance in five health outcomes and 25 determinants of health. The four health outcomes we used for our analysis are: poor or fair health, poor physical health days, poor mental health days, and low birthweight [5]. We obtained the first three health outcome measures from the BRFSS SMART, and the last from the NVSS Birth Records. Poor or fair health is based on the question, “Would you say that in general your health is…” with the response categories “excellent,” “very good,” “good,” “poor,” or “fair.” We treated respondents missing this variable (less than 1% of the sample) as not poor or fair. Poor physical health days is based on the question “Now thinking about your physical health, which includes physical illness and injury, for how many days during the past 30 days was your physical health not good?” and poor mental health days “Now thinking about your mental health, which includes stress, depression, and problems with emotions, for how many days during the past 30 days was your mental health not good?” For both of these variables we calculated the proportion of poor physical (mental) health days for each respondent by dividing the reported number of poor physical (mental) health days by 30. For respondents missing these variables (less than 2% of the sample), we assigned the average number of days per month in poor physical (mental) health across counties. We defined low birthweight as all live births weighing less than 2500 grams.

#### Group characteristics

We used education, sex, and race, as the group characteristics for measuring inequalities in each of the four health outcome variables. Education is either mother’s education (for low birthweight) or the respondent’s education (for the other three health outcomes). It is in four groups (less than high school, high school graduation, some college, and college graduation). We assigned respondents missing education information (0.39% of the BRFSS SMART sample) to “high school graduation” as the health of the respondents missing education information most closely resembled that of the respondents with high school graduation. Among the four education groups, only the lowest education group had less than 50 respondents or births in some counties (7 counties in BRFSS SMART and 3 counties in NVSS Birth Records), for which we collapsed the two lowest groups into one. Sex is binary, with no missing information. Race is in three groups (white, black, and other). We assigned respondents missing race information (1.37% of the BRFSS SMART sample) to “other.” In counties where “black” and/or “other” had less than 50 respondents or births, we collapsed these groups into “other” (21 counties in the BRFSS SMART and 30 counties in the NVSS Birth Records). We substituted missing information and collapsed groups with small numbers so we could preserve as many observations and counties as possible in the analysis.

### Measures of inequalities

**Figure 1:**
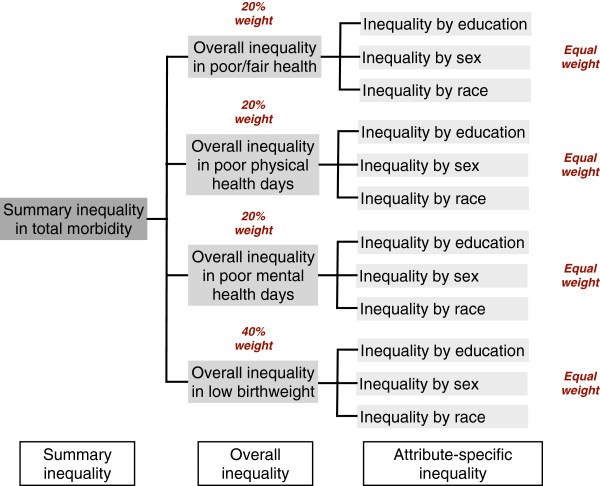
**Inequalities measured in each county.** For each county, we measured several inequalities. Attribute (group characteristic)-specific inequality is education-, sex-, or race-specific inequality in each of the four health outcomes (poor or fair health, poor physical health days, poor mental health days, and low birthweight). Overall inequality is the average of these three attribute-specific inequalities for each health outcome. Summary inequality in total morbidity is the weighted average of the overall inequalities across the four health outcomes: 20% each for poor or fair health, poor physical health days, poor mental health days, and 40% for low birthweight.

Measures of inequalities used in this study build on our previous work extending the inequality index proposed by Gastwirth [12,13]. For each county, we measured several inequalities and organized them as illustrated in Figure 1. *Attribute (group characteristic)-specific inequality* is education-, sex-, or race-specific inequality in each of the four health outcomes (poor or fair health, poor physical health days, poor mental health days, and low birthweight). *Overall inequality* is the average of these three attribute-specific inequalities for each health outcome. *Summary inequality* in total morbidity is the weighted average of the overall inequalities across the four health outcomes. We used the weights employed by the CHR: 20% each for poor or fair health, poor physical health days, poor mental health days, and 40% for low birthweight. We adjusted for age, using the US 2000 standard population, to calculate these measures of inequalities.

We calculated attribute-specific inequality using the index proposed by Gastwirth [12]. It calculates, for each group characteristic (e.g., education), the gap between the health of the healthiest group (e.g., the “college graduation” group) and the health of the rest of the groups (e.g., the “less than high school,” “high school graduation,” and “some college” groups). This calculation of the gap is sensitive to the group size but insensitive to the number of groups included. Values of attribute-specific inequality range between zero and one (0 ≤ and <1). Zero means all groups have the same health outcomes, thus, no inequality, while a value close to one suggests a greater gap between groups, hence, greater inequality. The value 0.034 of attribute-specific inequality suggests that, to eliminate inequality, an additional 3.4% of the population from the less healthy groups need to improve their health to the level of the healthiest group. Interpretations of values of overall inequality and summary inequality are similar. For overall inequality, one should interpret the value as an average across the three attributes considered, and for summary inequality, as an average across the three attributes and the four outcomes considered.

The same degree of overall inequality can come from different combinations of attribute-specific inequalities. For example, in some counties education-specific inequality may be larger than sex- and race-specific inequality, while in other counties race-specific inequality may be larger than others. For this reason, we also computed the contribution of each attribute-specific inequality to the overall inequality (e.g., education-specific inequality over the sum of all attribute-specific inequalities).

### Analysis

#### Calculating attribute (group characteristic)-specific, overall, and summary inequalities

From the pooled 2008–2010 BRFSS SMART, we obtained the proportion of the observations in poor or fair health and the average proportion of poor physical and mental health days per month in each group of education, sex, and race in each county. From the pooled 2008–2010 NVSS Birth Records, we obtained the proportion of births that were low birthweight in each group of maternal education, sex, and race, in each county. All BRFSS SMART numbers were age-standardized using the 2000 US standard population. Numbers from the BRFSS SMART were weighted using the county weight supplied by the 2008, 2009, and 2010 BRFSS SMART.

Using these numbers, we calculated attribute-specific inequality for each of the three attributes, overall inequality for each of the four health outcomes, and summary inequality in each county as described above. For the ease of interpretation, for all inequality calculations, we converted the proportions of negative health outcomes to those of positive health outcomes (e.g., the proportion not in fair or poor health).

#### Examining variations in inequalities

First, to understand to what extent inequality varied across counties, we described the minimum, 25^th^ percentile, median, 75^th^ percentile, and maximum of summary inequality across the 93 counties. Second, to assess whether the range of inequality differed by health outcome, we reported the minimum, 25^th^ percentile, median, 75^th^ percentile, and maximum of overall inequality across the 93 counties for each of the four health outcomes. Finally, to examine if patterns of attribute contribution differed across counties and by health outcome, for each health outcome and each county, we ranked the percent attribute contribution from 1 to 3 (1 being the largest contributor and 3 being the smallest contributor). We followed an ad hoc rule to consider attribute contributions within 5% as the same. We then assigned each of the 93 counties for each health outcome to one of the following seven categories: (1) education was the top contributor, (2) sex was the top contributor, (3) race was the top contributor, (4) education and race were tied as top contributors, and (5) race and sex were tied as top contributors, (6) education and sex were tied as top contributors (7) all three attributes were tied as top contributors.

We used Stata 11 [14] and Excel for all analyses.

## Results

**Figure 2:**
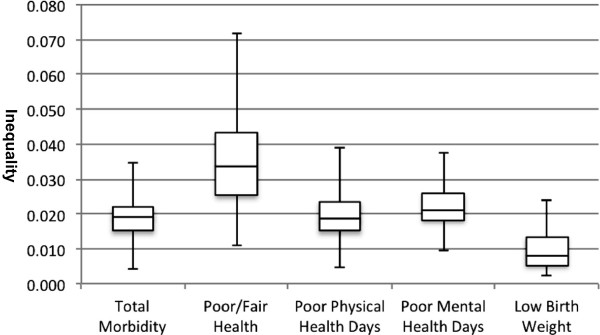
**The minimum, 25**^**th **^**percentile, median, 75**^**th **^**percentile, and maximum of summary inequality in total morbidity and overall inequality in each of the four health outcomes across 93 counties. **Data sources: A pooled 2008, 2009, and 2010 Behavioral Risk Factor Surveillance System (BRFSS) Selected Metropolitan/Micropolitan Area Risk Trends (SMART) and a pooled 2008, 2009, and 2010 United States Birth Records from the National Vital Statistics System (NVSS). Overall inequality in each of the four health outcomes is the average of these three attribute-specific (i.e., education-, sex, or race-specific) inequalities for each health outcome. Summary inequality in total morbidity is the weighted average of the overall inequalities across the four health outcomes: 20% each for poor or fair health, poor physical health days, poor mental health days, and 40% for low birthweight.

The summary inequality ranged from 0.004 (Minnehaha county, South Dakota) to 0.034 (Philadelphia county, Pennsylvania) (Figure 2, Table 1, and Additional file 1: Appendix 1). The average summary inequality of 0.019 across the 93 counties means that, to eliminate inequality in morbidity in a county, on average across the four health outcomes and the three attributes considered, an additional 1.9% of the population from the less healthy groups need to improve their health to the level of the healthiest groups. The sample size of the county was not associated with its summary inequality, suggesting little spurious influence of the sample size of the county on the magnitude of the summary inequality (Additional file 1: Appendix 2). Compared to Philadelphia county, which showed the largest summary inequality, Minnehaha county, which had the smallest summary inequality, exhibited smaller attribute-specific inequalities in all three attributes and four health outcomes (Table 1). Education had the largest contribution to overall inequalities in all four health outcomes in both of these counties, except overall inequality in low birthweight in Philadelphia county, where race made a slightly greater contribution (46.3%) than education (42.2%).The range of inequality differed by health outcome. Inequality in poor or fair health had the widest range (0.011-0.072) and the highest median (0.034) among overall inequalities of the four health outcomes (Figure 2). Overall inequality in poor physical health days and poor mental health days had similar ranges (0.005-0.039, 0.010-0.038, respectively) and medians (0.018, 0.021, respectively) while overall inequality in low birthweight had the narrowest range (0.002-0.024) and lowest median (0.008).

**Table 1:** Three counties with the smallest and largest summary inequality in total morbidity

	**Counties with the smallest summary inequality**			**Counties with the largest summary inequality**		
	**Minnehaha, SD**	**Douglas, CO**	**Douglas, NE**	**Aiken, SC**	**Berkeley, SC**	**Philadelphia, PA**
Summary inequality in total morbidity	0.004	0.007	0.009	0.030	0.030	0.034
Overall inequality						
Poor or fair health	0.020	0.013	0.026	0.060	0.052	0.068
Attribute-specific inequality (contribution) Education	0.044 (71.2%)	0.018 (45.7%)	0.056 (71.1%)	0.104 (57.2%)	0.114 (72.7%)	0.136 (66.5%)
Sex	0.014 (22.5%)	0.010 (24.9%)	0.005 (6.01%)	0.040 (22.1%)	0.015 (9.3%)	0.024 (11.7%)
Race	0.004 (6.3%)	0.011 (29.5%)	0.018 (22.8%)	0.038 (20.7%)	0.028 (18.1%)	0.044 (21.8%)
Poor physical health days	0.008	0.005	0.011	0.031	0.039	0.028
Attribute-specific inequality (contribution) Education	0.021 (92.1%)	0.006 (45.2%)	0.032 (94.8%)	0.058 (62.7%)	0.083 (71.2%)	0.052 (61.5%)
Sex	0.001 (2.7%)	0.007 (50.0%)	0.000 (0.0%)	0.028 (29.6%)	0.014 (12.2%)	0.026 (30.8%)
Race	0.001 (5.2%)	0.001 (4.8%)	0.002 (5.2%)	0.007 (7.7%)	0.019 (16.6%)	0.007 (7.8%)
Poor mental health days	0.014	0.010	0.016	0.026	0.035	0.032
Attribute-specific inequality (contribution) Education	0.027 (63.1%)	0.013 (29.2%)	0.022 (46.1%)	0.035 (45.2%)	0.052 (49.7%)	0.051 (53.7%)
Sex	0.015 (34.8%)	0.017 (37.6%)	0.000 (0.0%)	0.040 (51.1%)	0.037 (35.1%)	0.034 (35.4%)
Race	0.001 (2.1%)	0.015 (33.2%)	0.026 (53.9%)	0.003 (3.7%)	0.016 (15.3%)	0.010 (10.9%)
Low birthweigtht	0.006	0.003	0.009	0.016	0.013	0.022
Attribute-specific inequality (contribution) Education	0.011 (64.7%)	0.003 (25.9%)	0.014 (47.5%)	0.021 (43.9%)	0.023 (61.7%)	0.028 (42.2%)
Sex	0.003 (15.4%)	0.006 (55.9%)	0.004 (13.6%)	0.013 (26.3%)	0.000 (0.5%)	0.008 (11.5%)
Race	0.003 (19.9%)	0.002 (18.2%)	0.011 (38.9%)	0.014 (29.8%)	0.014 (37.9%)	0.031 (46.3%)

Data sources: A pooled 2008, 2009, and 2010 Behavioral Risk Factor Surveillance System (BRFSS) Selected Metropolitan/Micropolitan Area Risk Trends (SMART) and a pooled 2008, 2009, and 2010 United States Birth Records from the National Vital Statistics System (NVSS).

Attribute (group characteristic)-specific inequality is education-, sex-, or race-specific inequality in each of the four health outcomes (poor or fair health, poor physical health days, poor mental health days, and low birthweight). Overall inequality is the average of these three attribute-specific inequalities for each health outcome. Summary inequality in total morbidity is the weighted average of the overall inequalities across the four health outcomes: 20% each for poor or fair health, poor physical health days, poor mental health days, and 40% for low birthweight. Values of attribute-specific inequality range between zero and one (0 ≤ and <1). Zero means all groups have the same health outcomes, thus, no inequality, while a value close to one suggests a greater gap between groups, hence, greater inequality. The value 0.034 of attribute-specific inequality suggests that, to eliminate inequality, an additional 3.4% of the population from the less healthy groups need to improve their health to the level of the healthiest group. Interpretations of values of overall inequality and summary inequality are similar. For overall inequality, one should interpret the value as an average across the three attributes considered, and for summary inequality, as an average across the three attributes and the four outcomes considered.

**Figure 3:**
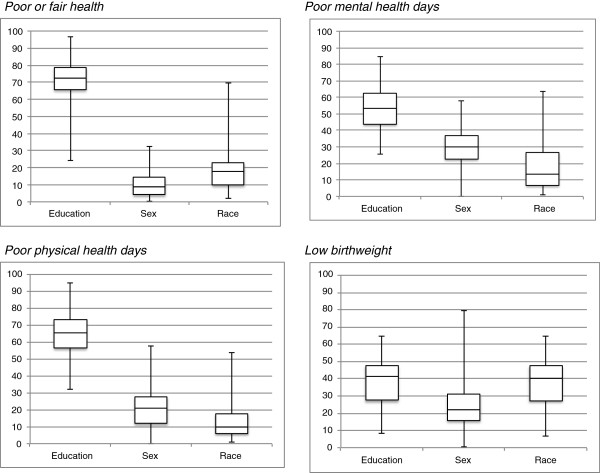
**The minimum, 25**^**th **^**percentile, median, 75**^**th **^**percentile, and maximum of attribute contributions (%) for overall inequality in each of four health outcomes in 93 counties. **Data sources: A pooled 2008, 2009, and 2010 Behavioral Risk Factor Surveillance System (BRFSS) Selected Metropolitan/Micropolitan Area Risk Trends (SMART) and a pooled 2008, 2009, and 2010 United States Birth Records from the National Vital Statistics System (NVSS).

Attribute contributions to overall inequality differed by health outcome (Figure 3). In more than 70% of the counties, education contributed most to overall inequality in poor or fair health, poor physical health days, and poor mental health days (Additional file 1: Appendix 3). The dominance of the contribution of education was less pronounced in overall inequality in low birthweight: in 38% of the counties education was the top contributor, and in another 38% of the counties race was the top contributor (Additional file 1: Appendix 3). While these general patterns of attribute contributions by health outcome emerged, the range of attribute contributions shown in Figure 3 suggests wide variation in attribute contributions across counties. In the large majority of counties, regardless of the health outcomes, the healthiest group within each attribute was: college graduation (the highest education group), male, and white.

## Discussion

A key message from this study is that no single group characteristic or health outcome represents the whole picture of health inequalities in a county. Our analysis showed that overall inequality in poor or fair health was on average greater and more variable across the 93 counties we examined, compared to overall inequalities in poor physical and mental health days and low birthweight. In a large majority of counties, education contributed most to overall inequality in poor or fair health and poor physical and mental health days, but the contribution of race was more pronounced for overall inequality in low birthweight. In sum, this study underscores the importance of examining multiple group characteristics and outcomes in a comparable manner in reporting health inequalities.

When adding the health inequality component to a population health report, one issue worth further attention is weighting [15,16]. In the measurement approach we used for this study, weighting becomes an issue for health outcomes and attributes. For health outcomes, we followed the CHR weighting procedure of a 40% weight to the health outcome of low birthweight and a 20% weight for each of the other three health outcomes to calculate total morbidity. These weights were derived from the literature and expert opinions [17,18]. For group characteristics, with no available guidance on this in the literature, we simply used equal weight for each group characteristic. Future work is necessary regarding what weights to use for different group characteristics, for what reasons, and who should decide these weights. It might be reasonable and even desirable to vary weights in different communities.

In addition to these methodological issues, data issues are of primary concern for health inequality reporting at a small jurisdictional level (and in low and middle income countries). The CHR measures five health outcomes, the four outcomes we used in our analysis plus premature death. We did not include premature death in our analysis because we were unable to locate county-level data on premature death by education. Along with education, sex, and race, income is one of the most common group characteristics with which health inequalities are examined [3]. We did not examine income-specific inequalities in our analysis because we were unable to obtain county-level data on low birthweight by income.

Another data issue is the problem of small numbers. This study showed the feasibility of measuring county-level inequalities in 93 counties, representing about 30% of the US population, using two publicly available, common data sources. These counties had relatively large sample sizes to allow subdivision of the data (e.g., 2 categories of self-rated health divided into 3 race groups within each county), but some counties did not have many persons belonging to minority groups. This was particularly the case for race, and we used three groups (white, black, and other), less refined than what is typically recommended in the literature [19,20].

Our strategies to deal with the problem of small numbers were threefold: merging data years, substituting missing information, and collapsing attribute groups. None of these strategies were unusual, but for the assessment of small area health inequalities to develop further, especially for regular reporting, more rigorous approaches should be established in the future. In this particular study, for example, we merged three years of data to increase the sample size. It is possible to merge many more years of data or include counties that appeared in only one of these years rather than all, as we restricted in this study. The decision regarding how many years of data to merge will be confined to how frequent the assessment of health inequalities takes place. If one intends to report health inequalities every other year, for example, one can only merge two years of data for timely reporting. In addition, practical considerations sometimes prohibit one from merging many years of data, for example, a coding or system change of data in certain years. Furthermore, virtually almost all data come with missing information. For this study, we employed ad hoc substitution strategies. For example, we assigned the average number of days per month in poor physical or mental health across counties because we reasoned that this would yield the least influence on the cross-county comparison. The use of county-specific average values would have been another option. Careful comparison of different imputation methods would reassure validity of this type of small-area analysis in the future. Finally, the attempt to solve small sample size issues by using less refined attribute groups is particularly problematic for race. For some counties, we needed to collapse an already rough three groups into two groups. When attributes required group collapsing, our feasibility analysis showed a possibility of spurious underestimation of inequality (results not shown). Given that the problem of small numbers will always be present when reporting health inequalities at a small jurisdictional level, in future work it will be helpful to provide margins of errors for inequality estimates whenever possible.

## Conclusions

While inequality reduction is frequently stated as one primary goal of population health improvement, inequality is often only measured for a single group characteristic or a single outcome measure. This study shows the feasibility of reporting inequalities for multiple group characteristics and outcomes at a small jurisdictional level. We encourage the use of such an approach more widely, as well as further research, data, and communication strategies to address some of the limitations we call attention to.

## Abbreviations

BRFSS: Behavioral risk factor surveillance system; CDC: Centers for disease control and prevention; CHR: County health rankings; MDGs: Millennium development goals; MMSAs: Metropolitan/Micropolitan statistical areas; NVSS: National vital statistics system; SMART: Selected metropolitan/micropolitan area risk trends.

## Competing interests

The authors declare that they have no competing interests.

## Authors’ contributions

YA collaborated in conceptualizing and designing the study, directly supervised data gathering and statistical analyses, and had primary responsibility for writing this article. AW collaborated in designing the study, gathering data, performing statistical analyses, and writing this article. DK collaborated in conceptualizing, designing the study, and writing this article. BB assisted to gather data, perform statistical analyses, and write this article. BR provided critical inputs in the study design, statistical analysis, and writing of the article. All authors read and approved the final manuscript.

## Supplementary Material

Additional file 1 Appendices. Click here for file
