# Supplementary material for: Inequalities in multiple health outcomes by education, sex, and race in 93 US counties: Why we should measure them all
Source: Int J Equity Health. 2014 Jun 13;13:47. doi: 10.1186/1475-9276-13-47 (PMC4070094; doi:10.1186/1475-9276-13-47)
Supplement: Additional file 1 — Appendices. [file 1475-9276-13-47-S1.docx]

Appendix 1. Summary inequality in total morbidity and overall inequality in each of the four health outcomes in 93 counties

Appendix 1. Summary inequality in total morbidity and overall inequality in each of the four health outcomes in 93 counties (continued)

Appendix 1. Summary inequality in total morbidity and overall inequality in each of the four health outcomes in 93 counties (continued)

Caption for Appendix 1

Data sources: A pooled 2008, 2009, and 2010 Behavioral Risk Factor Surveillance System (BRFSS) Selected Metropolitan/Micropolitan Area Risk Trends (SMART) and a pooled 2008, 2009, and 2010 United States Birth Records from the National Vital Statistics System (NVSS)

Attribute (group characteristic)-specific inequality is education-, sex-, or race-specific inequality in each of the four health outcomes (poor or fair health, poor physical health days, poor mental health days, and low birthweight). Overall inequality is the average of these three attribute-specific inequalities for each health outcome. Summary inequality in total morbidity is the weighted average of the overall inequalities across the four health outcomes: 20% each for poor or fair health, poor physical health days, poor mental health days, and 40% for low birthweight. Values of attribute-specific inequality range between zero and one (0 ≤ and <1). Zero means all groups have the same health outcomes, thus, no inequality, while a value close to one suggests a greater gap between groups, hence, greater inequality. The value 0.034 of attribute-specific inequality suggests that, to eliminate inequality, an additional 3.4% of the population from the less healthy groups need to improve their health to the level of the healthiest group. Interpretations of values of overall inequality and summary inequality are similar. For overall inequality, one should interpret the value as an average across the three attributes considered, and for summary inequality, as an average across the three attributes and the four outcomes considered.

Appendix 2. Summary inequality in total morbidity and sample size of all counties

Caption for Appendix 2

Data sources: A pooled 2008, 2009, and 2010 Behavioral Risk Factor Surveillance System (BRFSS) Selected Metropolitan/Micropolitan Area Risk Trends (SMART) and a pooled 2008, 2009, and 2010 United States Birth Records from the National Vital Statistics System (NVSS)

The sample size of the county was not associated with its summary inequality, suggesting little spurious influence of the sample size of the county on the magnitude of the summary inequality.

Appendix 3. Attribute contribution profiles among 93 counties

Caption for Appendix 3

Data sources: A pooled 2008, 2009, and 2010 Behavioral Risk Factor Surveillance System (BRFSS) Selected Metropolitan/Micropolitan Area Risk Trends (SMART) and a pooled 2008, 2009, and 2010 United States Birth Records from the National Vital Statistics System (NVSS)

For each health outcome and each county, we ranked the percent attribute contribution from 1 to 3 (1 being the largest contributor and 3 being the smallest contributor). We followed an ad hoc rule to consider attribute contributions within 5% as the same. We then assigned each of the 93 counties for each health outcome to one of the following seven categories: (1) education was the top contributor, (2) sex was the top contributor, (3) race was the top contributor, (4) education and race were tied as top contributors, and (5) race and sex were tied as top contributors, (6) education and sex were tied as top contributors (7) all three attributes were tied as top contributors.
